# The cascade of care for children and adolescents with HIV in the UK and Ireland, 2010 to 2016

**DOI:** 10.1002/jia2.25379

**Published:** 2019-09-09

**Authors:** Elizabeth Chappell, Hermione Lyall, Andrew Riordan, Claire Thorne, Caroline Foster, Karina Butler, Katia Prime, Alasdair Bamford, Helen Peters, Ali Judd, Intira J Collins, On behalf of the Collaborative HIV Paediatric Study Steering Committee, Katja Doerholt, Nigel Klein, Esse Menson, Paddy McMaster, Fiona Shackley, Julia Kenny, Delane Shingadia, Sharon Storey, Gareth Tudor‐Williams, Anna Turkova, Steve Welch, Claire Cook, Siobhan Crichton, Donna Dobson, Keith Fairbrother, Diana M Gibb, Lynda Harper, Marthe Le Prevost, Nadine Van Looy, Kate Francis, A Walsh, L Thrasyvoulou, S Welch, N Laycock, J Bernatoniene, F Manyika, G Sharpe, P Lewis, S Welch, B Subramaniam, L Hutchinson, P Ward, K Sloper, K Fidler, R Hague, V Price, M Clapson, J Flynn, A Cardoso, M Abou, N Klein, D Shingadia, P Ainsley‐Walker, P Tovey, D Gurtin, JP Garside, A Fall, S Yeadon, S Segal, C Ball, S Hawkins, M Dowie, S Bandi, E Percival, M Eisenhut, PK Roy, C Kavanagh, P McMaster, C Murphy, J Daniels, Y Lees, F Thompson, B Williams, L Cliffe, A myth, S Southall, H Freeman, K Fidler, S Christie, A Gordon, D Rogahn, S Harris, L Hutchinson, A Collinson, L Hutchinson, L Jones, B Offerman, M Greenberg, C Benson, A Riordan, R O'Connor, N Brown, L Ibberson, F Shackley, SN Faust, J Hancock, K Doerholt, M Sharland, S Storey, S Gorman, EGH Lyall, C Monrose, P Seery, G Tudor‐Williams, E Menson, J Broomhall, L Hutchinson, D Scott, J Stroobant, A Bridgwood, P McMaster, J Evans, E Blake, A Yannoulias, M O'Callaghan

**Affiliations:** ^1^ MRC Clinical Trials Unit at UCL London UK; ^2^ St Mary's Hospital Imperial College NHS Healthcare Trust London UK; ^3^ Alder Hey Children's NHS Foundation Trust Liverpool UK; ^4^ UCL Great Ormond Street Institute of Child Health London UK; ^5^ Paediatric Department Our Lady's Children's Hospital Crumlin Ireland; ^6^ St George's University Hospitals NHS Trust London UK; ^7^ Great Ormond Street Hospital for Children NHS Foundation Trust London UK

**Keywords:** HIV care continuum, paediatric, adolescents, cohort studies, UK and Ireland, retention in care

## Abstract

**Introduction:**

The UNAIDS 90‐90‐90 targets for the cascade of care are widely used to monitor the success of HIV care programmes but there are few studies in children. We assessed the cascade for children and adolescents living with HIV in the national Collaborative HIV Paediatric Study (CHIPS) in the UK and Ireland.

**Methods:**

Utilizing longitudinal data from CHIPS we compared the cascade of care for 2010, 2013 and 2016. Among children diagnosed with HIV and not known to be lost to follow‐up at the start of each calendar year, we summarized the proportion in active paediatric care during that year (defined as having ≥1 clinic visit, CD4 or viral load measurement, or change to antiretroviral therapy (ART) regimen), and of these, the proportion on ART at last visit in that year. Among those on ART, the proportion with viral suppression (<200 copies/mL) and good immune status (WHO immunological stage none‐/mild‐for‐age) at last visit in the year were summarized. Among those in care in 2016, outcomes were compared by current age, place of birth (born abroad vs. UK/Ireland) and sex.

**Results:**

Of children in paediatric HIV care at the start of 2010, 2013 and 2016 (n = 1249, 1157, 905 respectively), the proportion in active care during that calendar year was high throughout at 97 to 99%. Of those in active care, the proportion on ART increased from 79% to 85% and 92% respectively (*p *<* *0.001). Among those on ART, the proportion with viral suppression and good immune status was stable at 83% to 86% and 85% to 88%, respectively, across the years. Among children in care in 2016, those aged ≥15 years were less likely to be virally suppressed (79% vs. 91%, *p *<* *0.001) or to have good immune status (78% vs. 94%, *p *<* *0.001) compared to younger children; there were no differences by place of birth or sex.

**Conclusions:**

Children and adolescents in the UK and Ireland national cohort had high retention in care. The proportion on ART increased significantly over time although there was no change in viral suppression or good immune status. Poorer outcomes among adolescents highlight the need for targeted support for this population.

## 1 Introduction

The HIV cascade of care is a model which outlines the steps from HIV infection that individuals must pass through to achieve viral suppression, with intermediate stages including diagnosis, linkage to care and antiretroviral therapy (ART) initiation, and is widely used to monitor the performance of HIV healthcare systems 1, 2, 3. In 2014 UNAIDS announced the 90‐90‐90 treatment targets, with the aim that by 2020, 90% of people living with HIV should be diagnosed, 90% of those diagnosed should be on treatment, and 90% of those on treatment should be virally suppressed 4. The cascade of care has also been extended to stages including quality of life, immune recovery, and other markers of well‐being beyond just viral suppression 5, 6.

To date, the majority of studies describing the cascade of care are among adults with HIV 1, 6, 7, with few studies in children. The majority of those published focus on children within the context of prevention of vertical transmission of HIV 8, 9, 10, or on adolescents and young adults living with HIV 11. As children with vertically acquired HIV will require lifelong treatment 12, maintaining long‐term viral suppression on ART is a key challenge 13, 14. In this study, we utilized longitudinal data from the national Collaborative HIV Paediatric Study (CHIPS) in the UK and Ireland to assess the cascade of care in children and adolescents in 2010, 2013 and 2016, following the recommendation of universal treatment in 2016 15.

## 2 Methods

All children aged <16 years diagnosed with HIV in the UK and Ireland are reported to the National Study of HIV in Pregnancy and Childhood (NSHPC) and have been followed longitudinally in CHIPS since 2000 (with retrospective collection back to 1996) 16. Data collected include ART, CD4 and viral load (VL) measurements, and are pseudo‐anonymized. Both studies have NHS Research Ethics approval.

The cascade of care was assessed for three calendar years: 2010, 2013 and 2016. In adult cascade studies the first stage is normally the estimated number of adults living with HIV 17, but there are no equivalent data on the estimated number of children living with HIV in the UK/Ireland due to the high proportion of children born abroad who migrated into the country; our cascade therefore begins with the denominator of those diagnosed with HIV in the UK/Ireland. Children were eligible for inclusion and defined as in care if, as of the 1 January of each calendar year of interest, they had been diagnosed with HIV, were aged <21 years and not known to have died, left the country, or transferred to adult care, and had not been lost‐to‐follow‐up (LTFU) (defined as no visit in the previous three years, or otherwise reported by their clinic). In sensitivity analysis, all children ever LTFU were included in the denominator until age 21, regardless of time since their last visit.

Characteristics at the start of each year were described, and the following cascade stages summarized: (i) the proportion in active HIV care (defined as a clinic visit, CD4 or VL measurement, or change to ART regimen) in that year; (ii) of those in active HIV care, the proportion on ART at their last visit in that year; (iii) of those on ART, the proportion virologically suppressed (with two thresholds used: VL <200 and <50 copies/mL) at their last visit in that year. We added a fourth cascade stage to indicate the immunological status of patients: (iv) of those on ART, the proportion with good immune status (defined as WHO immunological stage none‐/mild‐for‐age, corresponding to CD4 >30% for those aged <1 year, CD4 >25% for those one to three years, CD4 >20% for those three to five years, CD4 >350 cells/mm^3^ for those ≥5 years 18) at their last visit in that year. Children with no available VL/CD4 measurement during the year were considered to be unsuppressed/to have poor immune status, but in a sensitivity analysis we included only those children with a VL/CD4 measurement available. We also present the proportion of the total cohort in care at the start of each year that were virally suppressed and with good immune status at last visit, irrespective of their retention or ART status.

Being on ART was defined as any ART regimen including mono or dual therapy or combination ART (cART), with cART defined as any regimen containing ≥3 drugs from ≥2 classes (excluding 2 class regimens with an unboosted protease inhibitor (PI)), or ≥3 nucleoside reverse transcriptase inhibitors (NRTI) including abacavir.

Retention at each step of the cascade was compared over the three calendar years using chi‐squared tests. In addition, in order to assess the most up‐to‐date cascade in more detail, characteristics of those not on ART in 2016 were described, and we stratified retention across the cascade for 2016 by age at the start of the year (<5, 5 to <10, 10 to <15, ≥15 years), sex, and place of birth (born abroad vs. born in the UK/Ireland).

All analyses were conducted using Stata version 15 (College Station, TX, USA).

## 3 Results

Of children ever followed in CHIPS by the 1 January each year, 1249/1781 (70%), 1157/1982 (58%) and 905/2095 (43%) met the inclusion criteria for the 2010, 2013 and 2016 analyses respectively (Table 1). The proportion eligible decreased over time, mostly because of an increasing proportion having transferred to adult care (17%, 28% and 42% in 2010, 2013 and 2016 respectively), which occurred at a median [interquartile range, IQR] 17.7 [16.8, 18.5] years of age. There were three deaths between 2010 and 2012 among children in care, and no deaths between 2013 and 2016. Patient characteristics were broadly similar across calendar years (Table 2). The vast majority (≥98% across all calendar years) had acquired HIV vertically. Over time there was an increase in the proportion of those born abroad diagnosed prior to their arrival in the UK/Ireland (from 19% in 2010 to 28% in 2016, *p *=* *0.001). Among those born abroad, the time between arrival into the country and first HIV diagnosis was relatively stable at around 0.5 years in each of the calendar years. The median [IQR] age at start of each year increased from 11.8 [8.5, 14.3] years in 2010 to 14.4 [11.2, 16.4] years in 2016 (*p *<* *0.001), and the median [IQR] age at ART initiation decreased from 5.3 [1.6, 10.2] to 4.6 [1.0, 9.5] years respectively (*p *=* *0.035).

**Table 1:** Reason for exclusion from cascade analysis, by calendar year

	2010 (N = 1781)	2013 (N = 1982)	2016 (N = 2095)
Excluded from analysis	532 (30%)	825 (42%)	1190 (57%)
Died	111 (6%)	114 (6%)	114 (5%)
Moved abroad	69 (4%)	79 (4%)	84 (4%)
Transferred to adult care	297 (17%)	547 (28%)	884 (42%)
Lost‐to‐follow‐up	40 (2%)	44 (2%)	34 (2%)
>21 years	15 (1%)	41 (2%)	74 (4%)
Included in analysis	1249 (70%)	1157 (58%)	905 (43%)

N refers to total number diagnosed in the UK/Ireland by the beginning of each calendar year.

**Table 2:** Patient characteristics, by calendar year

	2010 (N = 1249)	2013 (N = 1157)	2016 (N = 905)	*p*‐value
	n (%) or median [IQR]			
Sex: female	645/1248 (52%)	604/1157 (52%)	483/902 (54%)	0.687
Place of birth: born abroad	638/1247 (51%)	613/1156 (53%)	471/904 (52%)	0.658
Previously diagnosed abroad	121/638 (19%)	139/613 (23%)	134/471 (28%)	0.001
Age at diagnosis among those born in the UK/Ireland, years (n = 609, 543, 433)^a^	1.0 [0.3, 2.7]	0.9 [0.3, 2.7]	0.8 [0.3, 2.7]	0.875
Age at diagnosis in the UK/Ireland among those born abroad, years (n = 638, 613, 471)^a^	6.8 [4.2, 9.7]	7.0 [4.2, 10.3]	6.5 [3.7, 9.9]	0.370
Time between arrival and first HIV diagnosis in the UK/Ireland among those born abroad, years (n = 487, 455, 367)^a^	0.5 [0.1, 1.5]	0.4 [0.1, 1.5]	0.4 [0.1, 1.2]	0.789
Mode of infection: vertically acquired	1183/1203 (98%)	1097/1113 (99%)	867/880 (99%)	0.913
Ethnicity: black African	991/1237 (80%)	930/1148 (81%)	716/894 (80%)	0.736
Age at ART initiation, years	5.3 [1.6, 10.2]	5.2 [1.3, 10.2]	4.6 [1.0, 9.5]	0.035
Age at start of year, years	11.8 [8.5, 14.3]	13.2 [10.2, 15.6]	14.4 [11.2, 16.4]	<0.001
Duration of follow‐up in CHIPS, years	6.1 [3.2, 9.5]	7.6 [4.3, 11.0]	9.0 [5.5, 12.3]	<0.001

ART, antiretroviral therapy.

a n corresponds to the number with available date of diagnosis and/or date of arrival in the UK/Ireland.

### 3.1 Cascade of care over time

The proportion of children retained across each stage of the cascade in each year is shown in Table 3. The proportion in active care ranged between 97% and 99% across the three calendar years (*p *=* *0.004). Of those in active care, the proportion on ART increased from 79% in 2010 to 85% in 2013 and 92% in 2016 (*p *<* *0.001), corresponding to 76%, 84% and 90% of all those in care respectively. Among those on treatment, the proportion virologically suppressed <200 copies/mL and the proportion with good immune status were relatively stable at between 83% to 86% (*p *=* *0.163) and 85% to 88% (*p *=* *0.366) in the 3 years respectively, although there was an increase in the proportion suppressed <50 copies/mL from 72% to 79% and 77% respectively (*p *=* *0.003).

**Table 3:** The cascade of care, by calendar year

	2010	2013	2016	*p*‐value
	n (%)			
In active care during year	1208/1249 (97%)	1142/1157 (99%)	886/905 (98%)	0.004
Among those in active care: on ART	953/1208 (79%)	975/1142 (85%)	819/886 (92%)	<0.001
Among those on ART: virologically suppressed <200 copies/mL	793/953 (83%)	840/975 (86%)	701/819 (86%)	0.163
Among those on ART: virologically suppressed <50 copies/mL	688/953 (72%)	767/975 (79%)	632/819 (77%)	0.003
Among those on ART: good immune status^a^	814/953 (85%)	854/975 (88%)	711/819 (87%)	0.366
Of total cohort: virologically suppressed <200 copies/mL	809/1249 (64%)	859/1157 (74%)	714/905 (79%)	<0.001
Of total cohort: virologically suppressed <50 copies/mL	695/1249 (56%)	777/1157 (67%)	640/905 (71%)	<0.001
Of total cohort: good immune status^a^	1025/1249 (82%)	996/1157 (86%)	769/901 (85%)	0.020
Sensitivity analysis				
Among those on ART with a VL available: virologically suppressed <200 copies/mL	793/931 (85%)	840/943 (89%)	701/787 (89%)	0.014
Among those on ART with a VL available: virologically suppressed <50 copies/mL	688/931 (74%)	767/943 (81%)	632/787 (80%)	<0.001
Among those on ART with a CD4 available: good immune status^a^	814/920 (88%)	854/942 (91%)	711/770 (92%)	0.026

ART, antiretroviral therapy; VL, viral load.

a Good immune status defined as WHO immunological stage none‐/mild‐for‐age, corresponding to CD4 >30% for those aged <1 year, CD4 >25% for those one to three years, CD4 >20% for those three to five years, CD4 >350 cells/mm^3^ for those ≥5 years.

When considering the total cohort in care, the proportion virologically suppressed <200 copies/mL increased significantly from 64% in 2010 to 79% in 2016 (*p *<* *0.001), and the proportion with good immune status increased slightly from 82% in 2010 to 86% in 2013 to 85% in 2016 (*p *=* *0.020).

In the first sensitivity analysis, where we included children LTFU, the proportion in active care decreased to 94%, 95% and 94% in 2010, 2013 and 2016 respectively (data not shown). In the second sensitivity analysis including only children on ART with a VL/CD4 measurement available, the proportions suppressed <200 copies/mL and with good immune status both increased over time, from 85% to 89% (*p *=* *0.014) and 88% to 92% respectively (*p *=* *0.026).

### 3.2 Cascade of care in 2016

Among children in active care in 2016, 92% (819/886) were on ART at their last visit in 2016, of whom 7% (60/819) were on a non‐cART regimen: 22 (37%) were on boosted PI monotherapy, 15 (25%) were on a boosted PI and integrase inhibitor, and 14 (23%) were on a boosted PI and 1 NRTI/non‐nucleoside reverse transcriptase inhibitor (NNRTI). Viral suppression <200 copies/mL was higher among those on cART (87%, 659/759) compared to those on non‐cART regimens (70%, 42/60) (*p *<* *0.001).

Of the 67 (8%) children in active care but not on ART at last visit in 2016, 24 (36%) had previously received ART but subsequently stopped for a median of 326 [110, 1224] days, with the most common reason being adherence difficulties (n = 13, 54%). Among this group of patients subsequently off ART, the median age at the beginning of 2016 was 15.7 [13.2, 18.0] years. The median CD4 count among those aged ≥5 years (n = 18/22 with a CD4 measurement available) was 409 [315, 611] cells/mm^3^; five had a CD4 count ≤350 cells/mm^3^ and one had severe immunosuppression with CD4 ≤200 cells/mm^3^. Of two children aged <5 years, one had a CD4% measurement available of 46%. The remaining 43 (64%) were ART naïve, with a median age at the beginning of 2016 of 12.5 [9.7, 14.8] years, and of whom 20 (47%) were born abroad. The median CD4 count was 668 [575, 821] cells/mm^3^ among those aged ≥5 years (n = 33/41 with a CD4 measurement available), and the two children aged <5 years had CD4% of 37% and 41%. Forty (93%) of the ART‐naïve group had CDC stage N or A classification, and 3 (7%) stage B.

**Figure 1:**
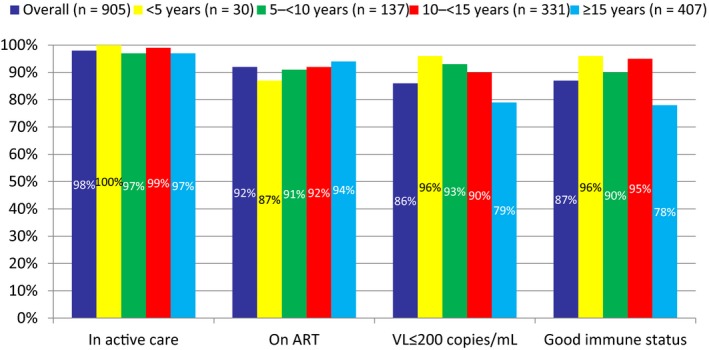
The cascade of care in 2016, overall and by age at start of 2016. Proportion with VL<200 copies/mL and with good immune status are among all on ART, assuming those with no VL and CD4 measurement available, respectively, did not meet the outcome.

The cascade of care by current age at start of 2016 is shown in Figure 1. Compared to younger children aged <15 years, those aged ≥15 years were less likely to be virally suppressed <200 copies/mL (79% vs. 91%, *p *<* *0.001) or have good immune status (78% vs. 94%, *p *<* *0.001), though there was no significant difference in the proportion in active care (*p *=* *0.252) or on ART (*p *=* *0.206). Similar trends were observed in the cascade for 2010 and 2013 (data not shown). A lower proportion of children born abroad had good immune status (84% vs. 89%, *p *=* *0.034), but there were no differences at other stages of the cascade of care, and there were no differences by sex (data not shown).

## 4 Discussion

In our national cohort of children and adolescents with HIV, the vast majority of whom acquired HIV vertically and had been in care for several years, we observed high levels of retention across the cascade in 2010, 2013 and 2016 and there were no deaths among children in paediatric HIV care between 2013 and 2016. This likely reflect the increase in the proportion on ART over calendar time and a decrease in the median age at initiation, consistent with treatment guideline changes in 2016 to recommend universal ART 15. While retention across other stages remained stable, there was a corresponding increase in the proportion of the total cohort who were virally suppressed and with good immune status. By 2016, 90% of the cohort were on ART, of which 85% were virologically suppressed, close to meeting the final two stages of the 90‐90‐90 targets. Of those children not on treatment, the majority were ART naïve with high CD4 counts and were clinically well, although one‐third had interrupted treatment. The total number in paediatric care decreased over time, as children aged and transferred to adult care.

Although there were no differences in retention in active care and on treatment, adolescents in our cohort had poorer outcomes on ART (viral suppression and good immune status) compared to younger children. In 2017, the UK reported to have met 90‐90‐90 target among adults aged ≥15 years 17; although an age‐disaggregated cascade was not provided, previous studies in the UK had reported poorer retention in the cascade among 15 to 24 year olds compared to older age groups 19. A global meta‐analysis also demonstrated poor adherence among adolescents 20, and another UK study from our group observed declining immunological outcomes with age among adolescents 21. This trend of poor outcomes has been observed to continue in young adults with perinatal HIV following transition to adult care, highlighting a key population who may need additional support to achieve the same level of cascade success 11, 22.

There are very few published studies of the paediatric cascade in high‐income countries, and to our knowledge this is one of the first comprehensive national assessments of the cascade of care, including children and adolescents with HIV across the UK and Ireland. One study of 525 children with perinatal HIV aged <18 years across the Netherlands in 2016 reported very high success rates with 96% on treatment, of whom 97% were virally suppressed ≤ 100 copies/mL 23. A study of 467 youth with horizontally acquired HIV aged 13 to 24 years in care across the USA during 2015 (of whom half had been diagnosed for less than 3 years) found 86% were engaged in care, of whom 98% were on ART, and of these 89% were suppressed <200 copies/mL 24. The proportion engaged in care in the USA study is substantially lower than in our cohort, this is possibly due to the difference in mode of infection and shorter duration since HIV diagnosis.

In our study we observed no effect of sex on the cascade. Children who were born abroad had comparable retention to those born in the UK/Ireland throughout the cascade, apart from a lower proportion having good immune status. However, it is important to note this is an unadjusted analysis, and that children who were born abroad were older when diagnosed with HIV and at ART start compared to UK and Ireland‐born children, which can limit the speed and extent of immune recovery 25. Our findings are consistent with a recent study of children across Europe, which reported no effect of migrant status in multivariable analyses on immune and virological outcomes on ART 26.

This study has a number of limitations. First, without being able to estimate the total number of children living with HIV in the UK/Ireland we were unable to estimate the proportion who had been diagnosed, corresponding to the first stage of the 90‐90‐90 targets. Although we have full coverage of those born with HIV in the UK, the number with HIV arriving in the UK or Ireland from abroad is difficult to assess. However, given that an increasing proportion of children born abroad were already diagnosed prior to arrival in the UK or Ireland 27, and that the median time between arrival and first HIV diagnosis in the UK or Ireland was short at six months, it is likely that most children living with HIV are captured. Second, we excluded those lost‐to‐follow‐up prior to each calendar year of interest from the denominator of the cascade. However, a very conservative definition of loss‐to‐follow‐up was used (not seen for >3 years, or otherwise defined by their clinic), making the assumption that those excluded were likely to have died or moved abroad, and results from sensitivity analyses were similar. Third, most adolescents with horizontally acquired HIV receive care in adult services in the UK and would therefore not be captured here. Finally, delay in reporting of data to CHIPS by clinics may have resulted in some individuals being incorrectly classified as not in active care.

## 5 Conclusions

Children and adolescents in the UK and Ireland had high retention in care throughout the calendar years assessed, close to meeting the second and third stages of the 90‐90‐90 targets. Provision of ART increased significantly over recent years although there was no change in the proportion with viral suppression or good immune status. Poorer outcomes among adolescents highlight the need for targeted support for this population. The cascade is an important tool to give an overview of the national picture, but more detailed study is required to understand what can be done to address leaks across all stages.

## Competing interests

CT has received funding from ViiV Healthcare (via PENTA Foundation) and AbbVie (plus other non‐commercial funders). AJ reports grants from Abbvie, Bristol Myers Squibb, Gilead, Janssen Pharmaceuticals and ViiV Healthcare through the PENTA Foundation, and from the Collaborative Initiative for Paediatric HIV Education and Research, Gilead Sciences, NHS England, Medical Research Council and PENTA Foundation outside the submitted work. KP reports funding from Gilead to attend conferences.

## Authors contributions

EC, IJC, CT and AJ designed the research study and wrote the first draft of the manuscript. All authors contributed to the collection of data, revising the manuscript and read and approved the final version.

### Funding

CHIPS is funded by the NHS (London Specialised Commissioning Group) and has received additional support from Abbott, Boehringer Ingelheim, Bristol‐Myers Squibb, GlaxoSmithKline, Gilead Sciences, Janssen and Roche. The MRC Clinical Trials Unit at UCL is supported by the Medical Research Council (https://www.mrc.ac.uk) programme number MC_UU_12023/26.

## Appendix 1

### 1.1

CHIPS Steering Committee: Hermione Lyall (Chair), Alasdair Bamford, Karina Butler, Katja Doerholt, Caroline Foster, Nigel Klein, Esse Menson, Paddy McMaster, Katie Prime, Andrew Riordan, Fiona Shackley, Julia Kenny, Delane Shingadia, Sharon Storey, Gareth Tudor‐Williams, Anna Turkova, Steve Welch. MRC Clinical Trials Unit at UCL: Intira J Collins, Claire Cook, Siobhan Crichton, Donna Dobson, Keith Fairbrother, Diana M Gibb, Lynda Harper, Ali Judd, Marthe Le Prevost, Nadine Van Looy. National Study of HIV in Pregnancy and Childhood: Kate Francis, Helen Peters, Claire Thorne.

Participating hospitals: Republic of Ireland: Our Lady's Children's Hospital Crumlin, Dublin: K Butler, A Walsh. UK: Birmingham Heartlands Hospital, Birmingham: L Thrasyvoulou, S Welch; Blackpool Victoria Hospital, Blackpool: N Laycock; Bristol Royal Hospital for Children, Bristol: J Bernatoniene, F Manyika; Calderdale Royal Hospital, Halifax: G Sharpe; Coventry & Warwickshire University Hospital, Coventry: P Lewis, S Welch; Derbyshire Children's Hospital, Derby: B Subramaniam; Derriford Hospital, Plymouth: L Hutchinson, P Ward; Middlesex: K Sloper; Eastbourne District General Hospital, Eastbourne: K Fidler; Glasgow Royal Hospital for Sick Children, Glasgow: R Hague, V Price; Great Ormond St Hospital for Children, London: M Clapson, J Flynn, A Cardoso, M Abou – Rayyah, N Klein, D Shingadia; Halliwell Children's Centre, Bolton: P Ainsley‐Walker; Harrogate District Hospital, Harrogate: P Tovey; Homerton University Hospital, London: D Gurtin; Huddersfield Royal Infirmary, Huddersfield: JP Garside; James Cook Hospital, Middlesbrough: A Fall; John Radcliffe Hospital, Oxford: S Yeadon, S Segal; King's College Hospital, London: C Ball, S Hawkins; Leeds General Infirmary, Leeds: M Dowie; Leicester Royal Infirmary, Leicester: S Bandi, E Percival; Luton and Dunstable Hospital, Luton: M Eisenhut; Handforth; Milton Keynes General Hospital, Milton Keynes: PK Roy; Newcastle General, Newcastle: T Flood, A Pickering; Liebeschuetz; Norfolk & Norwich Hospital, Norwich: C Kavanagh; North Manchester General, Manchester: P McMaster, C Murphy; North Middlesex Hospital, London: J Daniels, Y Lees; Northampton General Hospital, Northampton: F Thompson; Northwick Park Hospital Middlesex; B Williams; Nottingham QMC, Nottingham: L Cliffe, A Smyth, S Southall; Queen Alexandra Hospital, Portsmouth: H Freeman; Raigmore Hospital, Inverness:; Royal Alexandra Hospital, Brighton: K Fidler; Royal Belfast Hospital for Sick Children, Belfast: S Christie; Royal Berkshire Hospital, Reading: A Gordon; Royal Children's Hospital, Aberdeen: D Rogahn; Royal Cornwall Hospital, Truro: S Harris, L Hutchinson; Royal Devon and Exeter Hospital, Exeter: A Collinson, L Hutchinson; Royal Edinburgh Hospital for Sick Children, Edinburgh: L Jones, B Offerman; Royal Free Hospital, London: M Greenberg; Royal Liverpool Children's Hospital, Liverpool: C Benson, A Riordan; Royal Preston Hospital, Preston: R O'Connor; Salisbury District General Hospital, Salisbury: N Brown; Sheffield Children's Hospital, Sheffield: L Ibberson, F Shackley; Southampton General Hospital, Southampton: SN Faust, J Hancock; St George's Hospital, London: K Doerholt, K Prime, M Sharland, S Storey; St Luke's Hospital, Bradford: S Gorman; St Mary's Hospital, London: EGH Lyall, C Monrose, P Seery, G Tudor‐Williams; St Thomas’ Hospital (Evelina Children's Hospital), London: E Menson; Torbay Hospital, Torquay: J Broomhall, L Hutchinson; University Hospital Lewisham, London: D Scott, J Stroobant; Royal Stoke University Hospital, Stoke On Trent: A Bridgwood, P McMaster; University Hospital of Wales, Cardiff: J Evans, E Blake; Wexham Park, Slough: A Yannoulias; Whipps Cross Hospital, London: M O'Callaghan.
